# Loss of Individual and Social Identity: Consequences of Sexual Harassment of Iranian Nurses in the Workplace

**DOI:** 10.3389/fpsyg.2021.770859

**Published:** 2021-11-16

**Authors:** Maryam Zeighami, Parvin Mangolian Shahrbabaki, Mohammad Ali Zakeri, Mahlagha Dehghan

**Affiliations:** ^1^Nursing Research Center, Kerman University of Medical Sciences, Kerman, Iran; ^2^Department of Nursing, Faculty of Nursing and Mdiwifery, Kerman Branch, Islamic Azad University, Kerman, Iran; ^3^Department of Critical Care Nursing, Razi Faculty of Nursing and Midwifery, Kerman University of Medical Sciences, Kerman, Iran; ^4^Determinants of Health Research Centre, Non-communicable Diseases Research Center, Rafsanjan University of Medical Sciences, Rafsanjan, Iran

**Keywords:** sexual harassment, nurse, consequences, qualitative content analysis, Iran, lived experience

## Abstract

**Background:** Sexual harassment in the workplace is a common event with negative consequences for victims. Working conditions become unstable because of sexual harassment, and workplace insecurity causes psychological distress and physical problems, as well as a reduction in the quality of care. Therefore, the current study sought to investigate the effects of sexual harassment in the workplace on Iranian nurses.

**Materials and Methods:** This qualitative study used conventional content analysis with a descriptive-explorative approach to investigate the consequences of sexual harassment in Iranian nurses (*n* = 22). The purposeful sampling method was used. Semi-structured and in-depth interviews were used to collect data. Maximum diversity in terms of age, sex, work experience, level of education, marital status, and type of hospital and ward was observed in order to obtain rich information. The Guba and Lincoln criteria were used to improve the study’s trustworthiness and rigor, and the Graneheim and Lundman method was used to analyze the content.

**Results:** Two hundred and twenty-seventh number codes, one main category, four subcategories, and eighteen primary categories were extracted from the data in this study. The main category of “loss of individual and social identity: the consequences of sexual harassment in nurses” is divided into four subcategories: “psychological trauma,” “detrimental effects of work,” “physical problems,” and “disintegration of warm family relationships.” Sexual harassment had a greater psychological impact on victims.

**Conclusion:** Sexual harassment has a number of negative consequences for nurses’ personal and professional lives and can impose a significant burden on the healthcare system due to decreased productivity and loss of active labor. Therefore, it requires increased attention and focus.

## Introduction

Workplace violence (WPV) is a threat to employees’ safety, well-being, and health that is defined as incidents occurring in work-related situations in which employees are abused, threatened, or assaulted (McPhaul and Lipscomb, 2004). Nurses have been the most frequently targeted WPV worldwide, among all healthcare workers (Spector et al., 2014). Numerous studies have revealed that more than half of nurses have been subjected to verbal assault and approximately one-third have been subjected to physical assault (Ridenour et al., 2015). According to Yang et al. (2018), 94.6% of nurses reported a high prevalence of WPV, which can include verbal assault, physical assault, and sexual harassment.

Sexual harassment in nurses is an unacceptable international phenomenon and a serious problem; however, according to the literature, more than half of nurses (58.1%) have been subjected to at least one type of sexual harassment (Maghraby et al., 2020). However, sexual harassment in the clinical setting has been reported by 30–72% of registered nurses (Berry et al., 2012; Spector et al., 2014). As sexual harassment is reported rarely, the rate of sexual harassment in nursing may be much higher (Bondestam and Lundqvist, 2020). Sexual harassment is a form of sex discrimination, which puts either women or men at an unfair disadvantage. Gender-based violence has been used as a conceptual framework for understanding gender harassment, sexual harassment, sexual assault, date rape violence, and other forms of gendered violence (Latcheva, 2017). In other words, sexual harassment is a component of both actual and potential gender-based violence. Sexual coercion, as well as sexual abuse and assault, can all exist in different systems (Bondestam and Lundqvist, 2020). Sexual harassment of nurses is defined as inappropriate and bad words of sexual matters directed at a nurse, which directly or indirectly threaten a person’s ability to perform their tasks (Danielsen and DFAAPA, 2018).

Sexual harassment in the workplace is a worldwide problem. According to Kahsay et al. (2020), the frequency of sexual harassment among nurses ranges from 10 to 87.5%, with patients (46%) being the most common source. Tollstern Landin et al. (2020) discovered that in Tanzania, Africa, nearly 10% of nurses and nursing students were sexually harassed, with “sexual jokes and comment” (61.3%) and “unwelcome touching and hugging” (61.3%) being the most common forms of sexual harassment (48.4%). The study also revealed that sexual harassment in the workplace could be extremely dangerous, posing a serious job risk and stigma for nurses (Tollstern Landin et al., 2020). Sexual harassment is defined as a workplace problem that causes shame or humiliation, impairs the nurse’s ability to do the right thing, lowers the quality of work life, and endangers the well-being of victims. Furthermore, sexual harassment in the hospital setting can result in a reduction or loss of proper nursing care, affecting patient care (Nielsen et al., 2017; Kim et al., 2018). According to Ali and Ezz El Rigal (2019), sexual harassment in nursing students is prevalent in the clinical setting, affecting them physically, mentally, and in all aspects of their lives, and can lead to nurse’s burnout and poor practice. According to Kim et al. (2018) sexually harassed nurses experienced severe negative emotions. Sexual harassment has the potential to disrupt work and create an intimidating, hostile, or offensive environment. This can refer to a single incident or a series of incidents that were either intentional, unintentional, or coercive (Bondestam and Lundqvist, 2020).

Nielsen et al. (2017) demonstrated that sexual harassment is a multifaceted and complex phenomenon. Sexual harassment frequently causes a wide range of problems in the workplace. Workplaces rarely have guidelines or policies in place to manage and/or prevent sexual harassment or inappropriate sexual behavior, and this is frequently addressed on a temporary and sporadic basis (Nielsen et al., 2017). Many sexually harassed women, on the other hand, do not talk to anyone about their experiences and only a few report the most serious incidents to their workplace hierarchy or an official (Latcheva, 2017).

The reasons for sexual harassment of nurses are still unknown. Poor working conditions, hierarchical organizations, the normalization of gender-based violence, cultural context, and a lack of active leadership have all contributed to sexual harassment (Bondestam and Lundqvist, 2020). Therefore, as a serious occupational risk, sexual harassment can cause psychological complications for nurses and reduce psychological complications for nurses and reduce patient care (Nielsen et al., 2017; Kim et al., 2018). As a result, more understanding of the consequences of sexual harassment in nurses is required in order to support and assist sexually harassed victims (Tollstern Landin et al., 2020). As an obvious part of the problems of organizational-professional culture, it is necessary to take basic measures to manage inappropriate sexual behaviors and to prevent sexual harassment among nurses (Nielsen et al., 2017). As a result, understanding nurses’ experiences in such situations is critical. Identifying the effects of sexual harassment in the workplace on nurses can aid in the development of necessary interventions to reduce it (Najafi et al., 2018).

Since quantitative research methods cannot fully address such issues, qualitative research is frequently used to investigate phenomena in depth. As a result, it is necessary to investigate nurses’ experiences by analyzing their statements and feelings using a qualitative approach because the conditions of nurses in different countries differ in terms of sexual harassment experience. Therefore, the current study sought to investigate the effects of sexual harassment in the workplace on Iranian nurses. Nurse managers can use the findings of this study to identify complications and reduce sexual harassment in nurses.

## Materials and Methods

### Study Design and Setting

The current research is part of a larger project to develop and test the psychometric properties of a sexual harassment scale in Iranian nurses. This qualitative study used conventional content analysis in conjunction with a descriptive-exploratory approach. The term “content analysis” refers to a qualitative technique for analyzing written, verbal, or visual communication messages with the intent of describing phenomena. The purpose of this study was to examine both hidden and manifest content. This study was conducted in Kerman, the largest city in southeastern Iran.

### Sampling, Participant, and Data Collection

In the current study, sampling was continued until data saturation was reached, and information saturation was reached after interviews with 22 participants. Participants were chosen using purposeful sampling. Nurses working in various wards of hospitals affiliated with Kerman University of Medical Sciences (KUMS) and several private hospitals were interviewed. In addition, three of the participants were key informants. The interviews were held at pre-arranged times with the participants. To obtain rich and varied information, nurses with diverse and rich experiences were interviewed. Furthermore, various personal and occupational characteristics such as gender, age, marital status, level of education, work experience, position, type of hospital (public, private, educational) and wards in which they had work experience were chosen to provide a diverse range of information. There were 18 female and 4 male nurses (Table 1). The nurses’ ages ranged from 25 to 51 years, and their work experience ranged from 2 to 28 years. Some inclusion criteria were used to select participants. In this study, nurses with bachelor’s and advanced degrees and clinical experience were interviewed. The first researcher conducted individual semi-structured and face-to-face interviews. Table 2 shows some of the questions that demonstrate the consequences of sexual harassment. The interviews lasted 30–100 min, were audio recorded, and were handwritten verbatim. Samples were collected from September 2020 to April 2021.

**TABLE 1:** Characteristics of participants (*N* = 22).

**Participants characteristics**		**Number**
Sex	Female	18
	Male	4
Marital status	Single	7
	Married	12
	Divorced	3
Education level	Bachelor’s degree	15
	Master’s degree	6
	Ph.D.	1
Type of employment	Contract recruiters-1^*a*^	4
	Contract recruiters-2^*b*^	5
	Hired	13
Position	Nurse	14
	Head nurse	2
	Supervisor	3
	Faculty member	3
Hospital type*	Educational-governmental	20
	Non-educational-governmental	1
	Private	9
Type of ward*	Emergency	11
	Medical	8
	ICU	7
	Operation room	6
	CCU	5
	General surgery	4
	Nursing management office	3
	Orthopedics	3
	Psychiatry	3
	Pediatrics	2
	Gastroenterology	2
	Burn	1
	Dialysis	1

*^a^It is obligatory to work for government for 2 years at a lower rate of pay.*

*^b^Annually contracted with payment less than hired nurses.*

*Some participants had work experiences in different wards and hospitals.

**TABLE 2:** Example of questions.

**Questions**
1. Could you please tell me about your experience regarding sexual harassment in the workplace?
2. How did you feel and react when you were sexually harassed in the workplace?
3. What effect has sexual harassment in the workplace had on your physical and mental health?
4. How has sexual harassment in the workplace affected your career and work?
5. How has workplace sexual harassment affected your personal and family life?

### Ethical Consideration

The KUMS Ethics Committee approved all of the processes and procedures used in the study (Ethics Code: IR.KMU.REC.1399.353). First, the objectives of the study, the method of data collection and recording, the roles of the researcher and the participants, and the observance of privacy and confidentiality of the data were explained orally to the participants, who were assured that they could withdraw from the study at any time. The researcher obtained written consent from the participants before inviting them to participate in the study.

### Data Analysis

The following concepts are considered important when performing conventional qualitative content analysis: unit of analysis, meaning unit, condensation, code, category, and theme. The unit of analysis is the foundation of qualitative content analysis (Graneheim and Lundman, 2004). Each interview was treated as a separate unit of analysis in this study. The text was then divided into meaning units. Table 3 provides an example. The meaning units were condensed while keeping the theme in mind. The condensed meaning units were then labeled, and subcategories were created. The next step was to develop the categories that were characteristic of qualitative content analysis. The main category of “loss of individual and social identity: the consequences of sexual harassment in nurses” was obtained in the current study (Table 4). The analysis lasted from September 2020 to July 2021.

**TABLE 3:** Example of qualitative content analysis process.

**Main category**	**Categories**	**Subcategories**	**Code**	**Condensation**	**Meaning unit**
Loss of individual and social identity: consequences of sexual harassment of Iranian nurses in the workplace	Psychological trauma	Mental disorder	Nurse’s with depression due to sexual harassment	Physician abuse of room emptiness and nurse sexual harassment and nurse with depressive disorder	When the examination of the patients was finished, I wanted to leave, but the resident told me to stay, and he immediately closed the door and hugged me, kissing and touching my body. I was terrified, and my entire body shook. Following this incident, I became isolated and quiet. I cried all the time until I went to see a psychiatrist, who prescribed me antidepressants. I could not get this problem out of my head.
	Detrimental effects of work	Decreased quality care	Nurse’s medical error due to loss of concentration caused by sexual harassment	Touching and sexual harassment of a nurse by a resident in an empty ward, loss of concentration of the nurse and giving the wrong medication to the patient	I was working the nightshift. I was preparing the patient’s medicine in the workroom when the resident came in, closed the door, and before I could react, he hugged and touched me tightly from behind. I felt terrible, lost my concentration, and gave the wrong medicine to the patient.
	Physical problems	Illness	Headache and muscle pains following sexual harassment in workplace	Severe pain in neck, shoulder and back muscles of nurse due to excessive contraction following sexual jokes of colleagues	I had been getting many headaches; my neck, shoulders, and back were painful. After reviewing all of the images and tests, the doctor concluded that I had no problem; I simply contracted my muscles excessively. He asked me the reason. Then I discovered that this happened during genital surgeries because men used to make many sexual jokes during the operation, and I became irritated and subconsciously contracted my muscles.
	Disintegration of warm family relationships	Cold emotional relationships	Cold relationships due to being informed about sexual harassment in workplace	Cold relations because the spouse was informed about sexual harassment in the workplace; he became nervous and blamed his wife	He was a coworker who was constantly texting and calling. I told my husband the story so that he would not be suspicious and I could solve my problem with his assistance. He became very nervous when he heard this and blamed me. Instead of offering comfort, he scolded and has remained cold with me ever since.

**TABLE 4:** Primary categories, subcategories, and main category extracted from qualitative content analysis.

**Main category**	**Categories**	**Subcategories**
Loss of individual and social identity: consequences of sexual harassment of Iranian nurses in the workplace	Psychological trauma	Resentment and irritation Stress and anxiety Mental rumination Anger Feelings of guilt and shame Feeling of hatred Feeling of fear and insecurity Feeling of emptiness and worthlessness Mental disorders
	Detrimental effects of work	Decreased quality of care Feelings of isolation from the outside world Shifts in attitudes toward nursing Job loss
	Physical problems	Changes in appetite Sleep disorders Illness
	Disintegration of warm family relationships	Being reprimanded by the husband Cold emotional relationships

### Trustworthiness

To describe trustworthiness, qualitative research typically employs four criteria: credibility, conformability, dependability, and transferability (Kyngas et al., 2020). In the current study, several methods were used to increase trustworthiness.

In the current study, the researcher attempted to create a good relationship with the participants by staying in the field for a long time (1 year) to collect and analyze data. In order to collect a more in-depth data, the researcher tried to select participants with different characteristics (maximum variation). We also referred to the participants again and made revisions after assessing each interview, if necessary, to clarify ambiguities and corroborate the extracted thoughts (member check). In addition, we engaged two experienced researchers to analyze and interpret data, and all extracted codes and categories were reviewed and accepted by the authors to boost credibility. During the research procedure, the research team designed and produced a mind map in order to improve data confirmability. The transcripts of several interviews, as well as the codes and extracted categories, were given to the two members of research team (expert in conducting qualitative research), who were asked to double-check the data coding process’ accuracy. The external observer approach was used in this study to analyze his possible comparable understanding with the researcher and seek for contradictory examples in order to achieve the dependability. As a result, the data were handed to two researchers (expert in conducting qualitative research), and the data’s dependability was confirmed based on the same understanding. To increase the data’s transferability and appropriateness, the research findings were supplied to two of the same samples as the current study’s samples who were not participants, and their opinions were asked, and a conceptual generalization was made based on the similarities. We also tried to describe all the steps of the research in detail.

### Findings

The concept of “loss of individual and social identity: the consequences of sexual harassment in nurses” was presented at the end of the content analysis process. There are 18 primary categories and a main category. After continuous comparative analysis, code condensation, 227 codes remained (Table 4).

### Main Category: Loss of Individual and Social Identity: Consequences of Sexual Harassment in Nurses

According to the participants’ experiences, the consequences of nurse’s sexual harassment in the workplace include four subcategories: “psychological trauma,” “detrimental effects of work,” “physical problems,” and “disintegration of warm family relationships.”

#### Psychological Trauma

The male and female participants’ experiences revealed that sexual harassment had destructive effects on the soul and psyche. There was resentment and irritation, stress and anxiety, mental rumination, anger, along with feelings of guilt, shame, hatred, emptiness and worthlessness, fear and insecurity as well as mental disorders among the study’s participants.

**A- Resentment and irritation**: Some male and female participants reported having a bad feeling, crying, and severe discomfort because of sexual harassment.

*“It was impossible for me to believe. It made me feel awful. I was outraged that he allowed himself to have this sexual look at someone who was the same age as his own granddaughter.” (Participant No. 8, a woman with four years of clinical work experience)*

**B- Stress and anxiety:** According to the majority of male and female participants, sexual harassment such as touching, hugging, and frequent phone calls disrupted the nurses’ practices, especially during night shifts, and caused severe anxiety about attending night work and certain places such as elevators.

*“The patient*’*s companion hugged and kissed me from behind while I was working. I felt awful for a while and was very worried. I was nervous and irritated, and I was under a lot of stress at night work.” (Participant No. 13, a woman with 8 years of clinical work experience)*

**C- Mental rumination:** Some male and female participants reported disturbing thoughts due to constant recall of annoying scenes, mental preoccupation due to constant harassment, and mental conflict due to annoying words.

*“After the sexual harassment I experienced at work, I cannot stop thinking about it, and I worry constantly that my husband will discover what happened and refuse to let me go back to work.” (Participant No. 7, a woman with 17 years of clinical work experience)*

**D- Anger**: Some male and female participants reported feelings of rage and anger after being touched, pushed, shown unwanted love affair, asked for an illicit relationship, or subjected to obscene language.

*“Despite knowing that I am married, my co-worker sent me a love letter and asked for a relationship. I was furious. Every muscle in my body trembled with fury and rage. What did he take me for?” (Participant No. 1, a woman with 2 years of work experience)*

**D- Feelings of shame and guilt:** Participants reported feeling of shame and embarrassment regarding colleagues knowing about harassment, being touched by a coworker, and hearing the patient use obscene language in front of others. They also felt guilty due to not reporting sexual harassment.

*“The patient*’*s companion began cursing me, extremely vulgar and obscene insults that were humiliating in front of the others. I was drenched in sweat because of my embarrassment. For a long time, I felt guilty and blamed myself.” (Participant No. 21, a man with 7 years of clinical work experience)*

**E- Feelings of hatred:** Some participants reported feelings of disgust and hatred of the workplace, especially mixed work environments, hatred of marrying a doctor, hatred of male patients, and hatred of oneself.

*“Because of the harassment I witnessed from my male coworkers, I have become pessimistic about all the men who work in the hospital, and I forbade my daughter from marrying a doctor.” (Participant No. 14, a woman with 28 years of clinical and educational experience)*

**F- Feelings of emptiness and worthlessness**: Participants in the study reported low self-esteem and feelings of worthlessness.

*“She kept texting me and asking for a relationship and it irritated me that he only saw me as someone to satisfy his sexual desires. I felt insignificant.” (Participant No. 6, a woman with 2 years of clinical work experience)*

**G- Feelings of fear and insecurity:** Some participants reported extreme fear of being at work, fear of being alone with a male colleague after harassment, fear of coworkers realizing sexual harassment and defamation, fear of the spouse being informed and preventing her from working, and workplace insecurity.

*“I was summoned to the doctor*’*s room. When I walked in, he closed the door and, as he guided me to a seat, he wrapped his hand around my waist and began massaging my side. I was so terrified that my entire body was shaking. This is a fear I have had for a long time.” (Participant 9, a woman with 4 years of clinical work experience)*

**H- Mental disorder:** Some participants reported mental disorders such as obsessive-compulsive disorder, posttraumatic stress disorder, depression, and hospitalization in a psychiatric hospital.

*“I felt terrible after the sexual harassment I endured. I felt helpless, tired, and depleted of energy, and I was diagnosed with depression and prescribed medication.” (Participant No. 10, a woman with 10 years of clinical experience)*

#### Detrimental Effects of Work

Sexual harassment has been shown to have negative effects on a person’s job and career, according to participant feedback. Participants in the study reported decreased quality of care, feelings of isolation from the outside world, shifts in attitudes toward nursing, and job loss.

**A- Decreased quality of care:** Some male and female participants reported reduced concentration, forgetfulness of some care tasks, medical errors, decreased effective communication and interaction with patients, deliberate failure to do some care tasks due to fear of being at the patient’s bedside, and refusal to go to the ward round.

*“The harassment I witnessed had a significant impact on my interaction with the patient. I tried to be nice to patients and talk to them whenever possible, but I lost my trust in both the patient and his companion. I was not interested to talk to the patient, so my interaction with him was limited.” (Participant No. 12, a woman with 4 years of clinical experience)*

**B- Feeling of isolation from the outside world:** Some study participants reported that this type of consequence included ignoring the nurse’s words and requests, giving her too many night and hard work shifts due to dissatisfaction with an illegitimate relationship, reporting sexual harassment to superiors, and changing her workplace against her will.

*“I reported the surgeon*’*s abusive behavior to the head nurse, thinking the problem would be resolved, but the next day the head nurse called and said I have been transferred to the emergency department. I enjoyed working in the operating room because I had a lot of experiences in this field.” (Participant 19, a woman with 25 years of work experience)*

**C- Shifts in attitudes toward the nursing profession:** Some male and female participants stated that this consequence during their studentship changed their attitudes and wanted to leave nursing profession. Some participants also stated that sexual harassment changed their views on nursing negatively.

*“I have a bad feeling about this profession because I have had some very unpleasant sexual experiences. Even after all these years, this vision has become ingrained in me, and I do not advise anyone to pursue a career in this field.” (Participant No. 2, a woman with 26 years of clinical work experience)*

**D- Job loss:** Some male and female participants reported that this consequence resulted in absenteeism, quitting and resignation.

*“When I was in my first year of work and experienced sexual harassment, I did not go to work for about 2 weeks, was absent, and wanted to resign.” (Participant No. 15, a woman with 23 years of work experience)*

#### Physical Problems

The female participants’ experiences revealed that sexual harassment caused physical problems in the form of involvement and disease in various body systems. The study’s participants got sick, lost appetite, and slept poorly.

**A- Changes in appetite:** Some study participants reported changes in appetite, anorexia and anorexia nervosa, as well as bulimia, which led to severe overweight.

*“I became anorexic as a result of the unsolicited messages my male colleague sent me, which took over my life.” (Participant No. 2, a woman with 26 years of clinical work experience)*

**B- Sleep disorders:** The experiences of some participants showed that this consequence was in the form of sleep disorders at night, lack of sleep and nightmares.

*“I had nightmares for a long time. I did not sleep at night; I did remember that face and that movement.” (Participant 18, a woman with 15 years of clinical work experience)*

**C- Illness:** Participants reported physical problems like hand and body tremors, increased heart rate, paleness, headaches, muscle spasms and aches, back and shoulder pain, gastro intestinal ulcers, irritable bowel syndrome, hemorrhoids, and sleeping pill addiction.

*“I developed physical problems and irritable bowel syndrome after being sexually harassed at work. Migraine headaches became extremely severe, and I had a headache for several days.” (Participant No. 12, a woman with 4 years of clinical work experience)*

#### Disintegration of Warm Family Relationships

The female participants’ experiences revealed that one of the consequences of sexual harassment was the breakdown of family life. The study’s participants reported cold family relations and reprimands from their husbands.

**A- Being reprimanded by the husband:** According to the experiences of some study participants, this type of consequence took the form of being scolded, prejudiced, and restrained by the spouse.

*“When I told my husband about the sexual harassment, I expected consolation, but it was the opposite. He treated me coldly, he is angry at me, he blames me, he constantly interrogates me, and I have to tell him where I am going, what I am doing, and who I spoke with on the phone.” (Participant No. 7, a woman with 17 years of work experience)*

**B- Cold emotional relationships:** Some study participants reported negative effects on married life, comparison of their spouse’s behaviors with deceptive love expressions, and the cancelation of marriage.

*“I am cold with my husband. I am comparing my husband with that man. My husband does not say such words to me, and he does not say I love you once a day, but this man is always caring about me*.” *(Participant No. 1, a woman with 2 years of experience)*

## Discussion

This qualitative study aimed to understand how Iranian nurses deal with sexual harassment. This study highlights the need for a better understanding of sexual harassment in the nursing profession. The current study revealed that nurses faced many negative feelings and consequences of sexual harassment at workplace. Despite some studies in Iran on WPV among nurses, this is the first qualitative study on the negative consequences of sexual harassment among Iranian nurses. The analysis of nurse’ experiences revealed four main themes: psychological trauma, detrimental effects of work, physical problems, and disintegration of warm family relationships.

### Psychological Trauma

This refers to the psychological problems nurses face because of the negative consequences of sexual harassment in the workplace. Several studies, including this one, have found that sexual harassment can have negative psychological effects such as mental health disorders (Nielsen et al., 2010; Ali and Ezz El Rigal, 2019), sadness, shame, and anger (Tollstern Landin et al., 2020). Psychological imbalances can cause severe negative emotions and fear in nurses (Nielsen et al., 2017; Kim et al., 2018). Nielsen et al. (2010) discovered a correlation between sexual harassment and mental health in nurses (Nielsen et al., 2010). According to Ali and Ezz El Rigal (2019), sexual harassment of nursing students had an impact on their physical and mental health, and more than half of nurses complained of mental disorders, with a significant percentage of them complaining of insomnia. According to Tollstern Landin et al. (2020), victims of sexual harassment were upset when they returned to work and felt ashamed, angry, and depressed, which could have a negative psychological impact on nurses. According to Kim et al. (2018) sexually harassed nurses were unable to maintain a psychological balance, experienced severe negative emotions, and feared the consequences and long-term effects of sexual harassment. Nielsen et al. (2017) discovered that sexual harassment caused fear, feelings of insecurity and disability in nurses and healthcare workers. These findings show that sexual harassment of nurses, regardless of the type of society or culture, has had devastating psychological effects on nurses, and that basic measures must be taken to reduce and eliminate this problem. To reduce and treat the consequences of sexual harassment, it is recommended that nurses’ psychological problems be monitored and that initial interventions such as professional psychological counseling be implemented.

### Detrimental Effects of Work

Sexual harassment in the workplace has a negative impact on one’s job. According to the current study’s findings, sexual harassment is associated with factors that contribute to burnout, such as loss of occupational motivation (Tollstern Landin et al., 2020), job dissatisfaction (Nielsen et al., 2010; Ali and Ezz El Rigal, 2019; Maghraby et al., 2020), loss of job and negative job effects (Gabay and Shafran Tikva, 2020; Maghraby et al., 2020), problems with routine work processes (Nielsen et al., 2017), and negative effects on patient care (Kim et al., 2018; Gabay and Shafran Tikva, 2020). Tollstern Landin et al. (2020) demonstrated that sexual harassment could be a serious job risk and stigma for nurses in Tanzania, Africa, and an important factor in losing occupational motivation (Tollstern Landin et al., 2020). Similarly, Ali and Ezz El Rigal (2019) discovered that sexual harassment had an impact on nursing students’ job satisfaction (Ali and Ezz El Rigal, 2019). According to Maghraby et al. (2020), sexual harassment was associated with lower job satisfaction, which was the main complaint reported by more than half of the sexually harassed nurses (64.2%). Sexual harassment may also lead to nurses’ intention to leave their jobs, negatively affecting the nurses’ workforce (Maghraby et al., 2020). Nielsen et al. (2010) discovered a correlation between sexual harassment and job satisfaction (Nielsen et al., 2010). According to Hutagalung and Ishak (2012), sexual harassment reduces job satisfaction and increases job stress, and sexual harassment can be a predictor of job satisfaction and job stress. According to Nielsen et al. (2017), sexual harassment frequently leads to work-related problems such as long-term leave and resignation in nurses and healthcare workers. According to Gabay and Shafran Tikva (2020), sexual harassment can reduce nursing care and cause nurses to quit the profession. Kim et al. (2018) also demonstrated that one of the issues reported by sexually harassed nurses was its impact on patient care. These findings indicate that the negative occupational effects of sexual harassment cause a wide range of problems for nurses. It is critical to focus on solutions to reduce the negative effects of sexual harassment in the workplace. The most important point, however, is to take steps to prevent sexual harassment of nurses, which should be considered by nurse managers at the community level.

### Physical Problems

Nurses have reported physical problems as a negative consequence of sexual harassment in the workplace. However, the current study’s findings indicate that some nurses were so affected by sexual harassment that they developed physical illnesses in addition to mental health problems. According to Ali and Ezz El Rigal (2019), sexual harassment of nursing students had an effect on their physical condition, with a significant percentage of them complaining of insomnia. According to Gabay and Shafran Tikva (2020), sexual harassment leaves people feeling unprotected, lonely, and alienated. Maghraby et al. (2020) discovered that sexual harassment caused physical complications in sexually harassed nurses such as headaches and fatigue.

The prevalence of violence and physical problems varies by region to region in the world. According to Spector et al. (2014), the Anglo region is particularly prone to any type of violence, with physical violence being the most common in psychiatric units and emergency departments. However, some of these differences could be attributed to nurses being more open about sexual harassment in this region. However, the type of violence varies depending on the situation and the source of the violence (Spector et al., 2014). It is obvious that nurses are at risk of violence and physical problems. More research is needed to find effective solutions to reduce physical problems of nurses. Nurse managers should prioritize the use of rules and effective treatment protocols, as well as comprehensive support, in order to prevent and reduce physical problems of nurses.

### Disintegration of Warm Family Relationships

Nurses participating in the current study reported the breakdown of warm family relationships as a result of workplace sexual harassment. According to Ali and Ezz El Rigal (2019), sexual harassment of nursing students affected their family relationships. Maghraby et al. (2020) demonstrated that sexual harassment had an impact on the personal lives of sexually harassed nurses. According to a review of the literature, studies have focused less on the effects of sexual harassment on nurses’ family relationships. One of the main reasons participants refused to disclose sexual harassment could be disintegration of family ties. Another reason is that similar studies are paying more attention to the prevalence of sexual harassment. The current study, on the other hand, focused on the negative consequences of sexual harassment. Another reason could be the different cultural contexts of different societies, as well as individuals’ sensitivity to reporting the negative consequences of sexual harassment. Some nursing students believed that the definition of sexual harassment was ambiguous, and they accepted sexual harassment as an unavoidable part of their job (Birks et al., 2018). Sixty percent of nurses have also acted irresponsibly to reports of sexual harassment (Ali and Ezz El Rigal, 2019).

Nurses require a variety of strategies to deal with the negative consequences of sexual harassment in the workplace, including increased knowledge and awareness (Tollstern Landin et al., 2020), the need to diagnose and differentiate sexual harassment (Nielsen et al., 2017; Kim et al., 2018), early preparation (Davis and Richardson, 2017), an emphasis on self-care (Kim et al., 2018), an emphasis on correct reporting (Birks et al., 2018), the prevention and management of inappropriate sexual behaviors (Nielsen et al., 2017), and the need for security systems and adoption of policies (Ali and Ezz El Rigal, 2019), effective coping and strategies in nursing (Kim et al., 2018), and support of sexually harassed victims (Tollstern Landin et al., 2020). Nurses require more knowledge of sexual harassment, and hospitals and medical schools should focus on their ability to increase nurses’ sexual harassment knowledge and awareness (Tollstern Landin et al., 2020). Assisting nursing students in effectively diagnosing and dealing with sexual harassment is a critical component of ensuring learning quality care during clinical practice, which can increase nurses’ sensitivity (Ali and Ezz El Rigal, 2019) and prevent some future clinical problems. This necessitates a quick and decisive reporting system (Kim et al., 2018).

The current study’s findings indicate that a training program for sexual harassment prevention in nursing, as well as a systematic reporting system, are required (Birks et al., 2018). To reduce this problem, we must establish WPV management teams as well as appropriate rules and regulations that can improve workplace safety and patient care quality. There are effective strategies to deal with nurse harassment, such as documenting each incident and initiating legal proceedings, condemning sexual abuse, demanding the severe punishment for perpetrators, and improving the image of nurses in the media. Health care facilities should adopt policies and strategies aimed at reducing harassment and creating a safe working environment for nurses (Ali and Ezz El Rigal, 2019). In addition to prevention measures, hospitals and medical schools should do everything possible to support and assist sexually harassed victims (Tollstern Landin et al., 2020).

A comparison of the current study’s findings with similar studies from other countries demonstrates the severity of sexual harassment of nurses. Different social, cultural, and moral contexts, differences in hospital policies and management, work environment and conditions, and differences in nurses’ knowledge about sexual harassment can all have an impact on the severity of the consequences of sexual harassment among nurses. However, nurse managers must take immediate and comprehensive action to address these consequences. We hope to learn about nurses’ experiences with sexual harassment and the negative consequences as a result of this study. Furthermore, it is hoped that a safe environment free of sexual harassment will be created for nurses.

## Limitations

Although the interview was conducted individually and the nurses were assured that the information and findings of the interview were confidential, the participants may not have disclosed all information in this regard due to the cultural sensitivity of sexual harassment in Iran. Given that the majority of the participants in this study were female, generalizing the findings to both sexes should be done with caution. In present study setting, the majority of nurses were female, with a minor number of male nurses. Therefore, totally the number of male nurse were less than the females. Although, the researchers attempted to interview as many male participants who had experienced sexual harassment as possible, we could not find more male nurses with experience of sexual harassment that want to participate in the study. May be the female gender of the interviewer or who identified them to us has influenced on their willingness. The current study’s findings were obtained from southeastern part of Iran. Due to the various cultural and ethnic differences in Iran, these differences should be taken into account in future studies. As many studies around the world have been used in this regard, current findings can extend beyond the cultural context of Iran. More research, however, is required to understand the negative consequences of sexual harassment in nurses.

## Conclusion

The study’s findings provide important and practical insights into nurses’ perceptions of sexual harassment in the workplace. According to the findings of this study, nurses face a wide range of problems in their personal and social lives as a result of the numerous and destructive negative effects and consequences of sexual harassment in the workplace. Nurses in the current study faced numerous challenges, including psychological trauma, detrimental effects of work, physical problems, and the disintegration of warm family relationships. To overcome these obstacles, they require effective strategies, comprehensive support, effective enforcement of laws and policies, and organizational measures to prevent and reduce sexual harassment, which nurse managers at the community level must consider. Nurse managers should be aware of a negative impact of sexual harassment on nurses. As a result, the need to prevent and reduce sexual harassment among nurses should be prioritized. These efforts aim to reduce the negative effects of sexual harassment on nurses while also improving health-care quality. However, more research is needed to understand the negative consequences of sexual harassment in nurses in various social, cultural, and moral contexts.

## Data Availability Statement

The raw data supporting the conclusions of this article will be made available by the authors, without undue reservation.

## Ethics Statement

The studies involving human participants were reviewed and approved by the Kerman University of Medical Sciences. The patients/participants provided their written informed consent to participate in this study.

## Author Contributions

MZ, PMS, and MD designed the study and collected the data. MZ, PMS, MAZ, and MD contributed to the study design, they provided critical feedback on the study and qualitative analysis, and inputted to the draft of this manuscript. MZ and MAZ wrote the manuscript. All authors have read and approved the final manuscript.

## Conflict of Interest

The authors declare that the research was conducted in the absence of any commercial or financial relationships that could be construed as a potential conflict of interest.

## Publisher’s Note

All claims expressed in this article are solely those of the authors and do not necessarily represent those of their affiliated organizations, or those of the publisher, the editors and the reviewers. Any product that may be evaluated in this article, or claim that may be made by its manufacturer, is not guaranteed or endorsed by the publisher.
